# Eplerenone improves kidney function and cardiac performance in obese male mice

**DOI:** 10.1111/dme.70154

**Published:** 2025-10-03

**Authors:** Ayman K. Banah, Bingxue Qi, Ryan Koh, Alexander Lawlor, Tianzhen Wang, Vishal Musale, Joanne Mains, Colin E. Murdoch, Li Kang

**Affiliations:** ^1^ Division of Diabetes, Endocrinology, and Reproductive Biology, School of Medicine University of Dundee Dundee, Scotland UK; ^2^ Nursing Sciences and Research, College of Nursing Umm Al‐Qura University Makkah Saudi Arabia; ^3^ Precision Molecular Medicine Center Jilin Province People's Hospital Changchun Jilin China; ^4^ Division of Cardiovascular Research, School of Medicine University of Dundee Dundee, Scotland UK

**Keywords:** cardio‐renal dysfunction, eplerenone, fibrosis, obesity

## Abstract

**Aims:**

Renin‐angiotensin‐aldosterone system (RAAS) activation mediates obesity‐associated cardiorenal dysfunction. Due to an incomplete understanding of the molecular mechanisms, the clinical use of RAAS antagonism in obesity‐associated cardiometabolic complications remains uncertain. The present study investigated the effects and associated molecular mechanisms of eplerenone, a selective mineralocorticoid receptor antagonist, in the heart and kidney of obesity.

**Methods:**

Male C57BL/6 mice were fed a high‐fat diet (HFD) for 12 weeks before receiving either vehicle or eplerenone treatments for 30 days while maintained on HFD. Cardiac function was measured by pressure–volume (PV) loop analyses. Renal function was assessed by renal morphology and serum creatinine concentrations. Chow‐fed mice were used as lean controls.

**Results:**

HFD feeding impaired cardiac and renal function in mice, evidenced by elevated blood pressures, stroke work, Tau, glomerular hypertrophy and tubular injury. Eplerenone treatment caused weight loss, which was attributed to a loss in fat mass. Moreover, eplerenone treatment ameliorated both HFD‐induced cardiac and renal dysfunction, including a decrease in stroke volume, cardiac output and stroke work; an increase in ejection fraction; and a decrease in glomerular hypertrophy, tubular injury, renal fibrosis and serum creatinine concentrations. Mechanistically, eplerenone decreased CD44 but increased RHAMM and TGF‐β expression in the heart. In contrast, eplerenone caused a decrease in the hyaluronan‐CD44/RHAMM pathway, TGF‐β, IL‐6 and phosphorylation of Akt and JNK in the kidney.

**Conclusion:**

Eplerenone treatment improved kidney function and cardiac performance in obese male mice. The potential link between the mineralocorticoid receptor and the hyaluronan‐CD44/RHAMM pathway is novel and has not been previously reported.

**What's new?**

- High fat diet feeding in obese male mice activates RAAS activity and elevates circulating aldosternone concentration.
- Eplerenone, a selective mineralocorticoid receptor antagonist, improves kidney function and cardiac performance in obese male mice.
- Eplerenone does so through novel mechanisms involving the hyaluronan‐CD44/RHAMM pathway, TGF‐β, IL‐6, phosphorylation of Akt and JNK in a tissue‐dependent manner.

## 1 INTRODUCTION

Obesity is a significant risk factor for the development of chronic kidney disease (CKD) and cardiovascular disease (CVD). Obesity contributes to both renal and cardiac dysfunction, including glomerular hyperfiltration, increased renal sodium reabsorption, heightened intraglomerular pressure, left ventricular hypertrophy and cardiomyopathy, through various pathophysiological mechanisms.^1^ One of the critical mediators in these processes is the activation of the renin‐angiotensin‐aldosterone system (RAAS).

In obesity, RAAS activity is upregulated, leading to elevated levels of angiotensin II (Ang II) and aldosterone.^2^ These hormones contribute to kidney damage by promoting inflammation, fibrosis and endothelial dysfunction. Ang II, in particular, stimulates the production of reactive oxygen species (ROS) and pro‐inflammatory cytokines, exacerbating renal injury and accelerating the progression of CKD.^3^ In addition, aldosterone contributes to renal pathology by inducing proteinuria and promoting sodium retention, thereby exacerbating hypertension. The elevated aldosterone levels seen in obesity can lead to maladaptive responses, such as glomerulosclerosis and tubulointerstitial fibrosis, which are hallmarks of CKD.^4^ Consequently, targeting the RAAS using mineralocorticoid receptor antagonists has been shown to ameliorate the detrimental renal pathology,^5^ thereby protecting renal function and improving outcomes in obese individuals.

In the context of cardiac dysfunction, increased aldosterone and mineralocorticoid receptor signalling contribute to adverse cardiac remodelling, morbidity and mortality.^6^ Mineralocorticoid receptor antagonists such as spironolactone and eplerenone have demonstrated efficacy in ameliorating these detrimental effects, showcasing the therapeutic potential for cardiovascular pathophysiology.^7^

RAAS overactivity has also been implicated in the pathogenesis of insulin resistance. Ang II impairs insulin signalling in skeletal muscle and adipose tissue by increasing oxidative stress and inflammation, which interferes with insulin‐stimulated GLUT4 translocation and downstream signalling pathways such as Akt phosphorylation, ultimately reducing glucose uptake.^8^, ^9^, ^10^ Similarly, aldosterone acting through the mineralocorticoid receptor disrupts insulin receptor signalling by promoting oxidative stress and serine phosphorylation of insulin receptor substrate‐1, and by stimulating inflammatory pathways in adipose and vascular tissues.^11^, ^12^ Consequently, pharmacological blockade of RAAS, particularly via mineralocorticoid receptor antagonists, has been shown to improve systemic insulin sensitivity and glycaemic control in both experimental and clinical models.^5^, ^13^ This highlights RAAS as a critical mediator at the intersection of renal, cardiovascular and metabolic disease, and underscores the broader therapeutic relevance of mineralocorticoid receptor antagonism in addressing complications of obesity and diabetes.

Eplerenone, a selective aldosterone receptor antagonist and a medication, is commonly prescribed to treat hypertension, heart failure and primary hyperaldosteronism. Eplerenone reduces proteinuria and slows the progression of CKD in patients with metabolic syndrome and obesity.^14^ By blocking the effects of aldosterone, eplerenone mitigates inflammation, fibrosis and oxidative stress within the kidney and reduces morbidity and mortality associated with heart failure with reduced ejection fraction (HFrEF),^15^ thus preserving renal and cardiac function.^16^, ^17^, ^18^ Despite the well‐established clinical benefits of eplerenone, its long‐term and dual effects in the heart and kidney of obesity and associated mechanisms of action remain unclear, which limits its further clinical application in obesity and obesity‐related complications such as type 2 diabetes mellitus.

In this study, we investigated the metabolic regulation and potential cardiorenoprotective effects of 30‐day eplerenone treatment in high‐fat diet (HFD)‐fed obese mice. We discovered novel cellular mechanisms by which eplerenone mitigates obesity‐related kidney pathology and myocardial remodelling, respectively.

## 2 MATERIALS AND METHODS

### 2.1 Mouse model and treatment regimen

Animal experiments were conducted in accordance with the UK Animals (Scientific Procedures) Act 1986 and were approved by the Animal Care and Use Committee of the University of Dundee. All procedures adhered to the guidelines of Directive 2010/63/EU of the European Parliament on the protection of animals used for scientific purposes. Mice were housed in an air‐conditioned room maintained at 22 ± 2°C with a 12:12‐h light–dark cycle. Mice had free access to food and water. Animal numbers varied across protocols due to differential attrition rates and the exclusion of data based on outlier analyses. Outliers were identified as values exceeding 1.5‐fold standard deviations from the group means. Sample sizes were power calculated based on prior data using the same method in the same laboratory. The exact sample numbers for each measurement were noted or presented as individual data points where possible.

#### 2.1.1 HFD‐induced obesity

Male C57BL/6J mice were purchased from the Charles River Laboratory. After one week of acclimatisation, mice at 7 weeks of age were fed a HFD (60% calories from fat, SDS 824054) for 16 weeks. Mice that were maintained on a chow diet (13% calories as fat, LabDiet 5001) were used as lean controls. At the end of the feeding period, mice underwent Pressure–volume (PV) loop analyses for the measurement of cardiac function. Thereafter, tissues including left ventricle and kidneys were collected for further analyses.

#### 2.1.2 Eplerenone treatment

In a separate cohort of mice, after 12 weeks of HFD feeding, mice were randomly assigned into two groups, HFD Vehicle or HFD Eplerenone. Eplerenone (WuXi App Tec, China, #107724‐20‐9) at 200 mg/kg body weight or vehicle (saline) was given orally once daily for 30 days. After 3 weeks of treatment, mice underwent an oral glucose tolerance test, followed by an insulin tolerance test a week later. PV loop analyses were then performed to assess cardiac function. Thereafter, tissues including the left ventricle and kidneys were collected for further analyses. The body weight of mice was monitored weekly throughout the study and daily during the eplerenone treatment. Body composition was measured before and after the treatment with vehicle or eplerenone using EchoMRI (Echo Medical Systems, TX).

### 2.2 Glucose and insulin tolerance tests

Mice were fasted for 5 h. An oral administration of 50% glucose (2 g/kg body weight) was given to mice, and blood glucose was monitored through tail bleeding for 2 h. Blood samples were obtained at 0, 10, 30 and 60 min, to monitor plasma insulin concentrations. For insulin tolerance test, intraperitoneal insulin injections were administered at 0.75 U/kg body weight. Blood glucose levels were monitored through tail bleeding for 60 min.

### 2.3 PV loop analysis

The cardiac function of experimental mice was assessed in real‐time using left ventricle PV loops. This was performed with an admittance catheter (1.2F, Transonic) connected to an ADV500 data acquisition system (Transonic) and visualized using LabChart (ADInstruments). Mice were anesthetized with 2% isoflurane (volume/volume) inhalation for 30 min, and body temperature was continuously monitored using a rectal thermometer probe. The PV catheter, which has both pressure and volume sensors, was initially inserted into the aorta via the carotid artery to measure arterial pressure. It was then advanced into the left ventricle to record pressure and volume signals under basal conditions and during inferior vena cava (IVC) occlusion, as previously described.^19^ IVC occlusion, the gold standard for load‐independent measurements of contractility (end‐systolic pressure–volume relationship, ESPVR) and compliance (end‐diastolic PV relationship, EDPVR), was achieved by temporarily blocking the return of blood to the heart. The hemodynamic data, both load‐dependent and load‐independent, were analysed using LabChart Pro 8 software (ADInstruments). After completing the experimental procedures, mice were euthanized by exsanguination under anaesthesia, and tissues were collected for further analyses.

### 2.4 Immunohistochemistry

Paraffin‐embedded left ventricular and kidney tissues were sectioned at 5 μm and mounted onto staining slides. Expression of hyaluronan, collagen and α‐SMA (smooth muscle actin) was assessed by biotinylated hyaluronan‐binding protein (AMS.HKD‐BC41, AMS Biotechnology), Picrosirius Red (Direct Red 80, Sigma 365548) and anti‐α‐SMA antibody (Cell Signalling 19245), respectively. Periodic acid‐Schiff (PAS) staining (#ab150680; Abcam) was performed to assess kidney morphology and tubular damage. The kidney cortex was evaluated for tubular epithelial cell vacuolar deformation/hypertrophy, tubular dilation, loss of brush border and cell lysis. The degree of tubular damage was scored using the system described by Haut et al.^20^ Slides were lightly counterstained with Mayer's haematoxylin for hyaluronan and α‐SMA staining. Images of distinct areas (10–12× per animal) were captured using an Axiovision microscope (Zeiss Axioscope, Germany) and quantified with ImageJ software. Image analysis was performed under blinded conditions.

### 2.5 Western blotting

The left ventricles and kidneys of mice were homogenized in lysis buffer containing protease and phosphatase inhibitors, as previously described.^21^ Protein concentrations were determined, and 20–40 μg of protein per sample was separated using 10% SDS‐PAGE gels. The proteins were detected using specific antibodies: TGF‐β (ab179695, 1:1000; Abcam), Phospho‐Smad2 (Ser465/467)/Smad3 (Ser423/425) (8828, 1:1000; Cell Signalling), Smad2/3 (3102, 1:1000; Cell Signalling), Phospho‐p38 MAPK (Thr180/Tyr182) (9211, 1:1000; Cell Signalling), p38 MAPK (9212, 1:1000; Cell Signalling), Phospho‐SAPK/JNK (Thr183/Tyr185) (9251, 1:1000; Cell Signalling), JNK (9252, 1:1000; Cell Signalling), pERK1/2 (4370, 1:1000; Cell Signalling), ERK (4695, 1:1000; Cell Signalling), pAKT (9271, 1:1000; Cell Signalling), AKT (9272, 1:1000; Cell Signalling), RHAMM/CD168(E7S4Y) (87129, 1:500; Cell Signalling), CD44 (AF6127; 1 μg/mL; R&D Systems), GAPDH (5174, 1:1000; Cell Signalling), beta‐tubulin (ab6046, 1:1000; Abcam) and Ponceau staining were used as loading controls as appropriate.

### 2.6 Quantitative real‐time PCR (qRT‐PCR)

Total RNA was isolated using TriPure reagent and subsequently reverse transcribed into cDNA with SuperScript™ II Reverse Transcriptase (ThermoFisher, 18064014). qRT‐PCR was performed to amplify target genes using the Veriti 96‐well Thermal Cycler (ThermoFisher). The following primer sequences were used:

*TNF‐α* (Forward: AAGAGTTCCCCAGGGACCTCT, Reverse: CCTGGGAGTAGATGAGGTACA), *IL‐1β* (Forward: TGCCACCTTTTGACAGTGAT, Reverse: GATTTGAAGCTGGATGCTCT), *IL‐6* (Forward: GAAAAGAGTTGTGCAATGGCAAT, Reverse: TTGGTAGCATCCATCATTTCTTTG), *IL‐10* (Forward: ACTGGCATGAGGATCAGCAG, Reverse: CTCCTTGATTTCTGGGCCAT), *BNP* (Forward: ACAGAAGCTGCTGGAGCTGA, Reverse: CCGATCCGGTCTATCTTGTG), *β‐MHC* (Forward: TATCGATGACCTGGAGCTGA, Reverse: AGTATTGACCTTGTCTTCCTC), *18S* (Forward: GCAATTATTCCCCATGAACG, Reverse: GGCCTCACTAAACCATCCAA) (Merck). Additionally, probes from ThermoFisher were used for *Col1a1* (Mm00801666_g1), *Col3a1* (Mm00802331_m1) and *18S* (Hs99999901_s1). Gene expression data were normalized to 18S and analysed using the 2^−ΔΔCT^ method.

### 2.7 Blood biochemistry

Serum creatinine levels were measured using a commercial kit (ab65340; Abcam). Plasma insulin concentration was measured using the Ultra‐Sensitive Rat Insulin ELISA kit (Crystal Chem, #90060). Plasma and tissue IL‐6 concentrations were measured using the IL‐6 Mouse ELISA Kit (ab100713; Abcam). Plasma aldosterone concentration was measured using the Aldosterone ELISA Kit (ab136933; Abcam). Absorbance of colorimetric reactions was measured using a Thermo Scientific Multiskan Spectrum plate reader.

### 2.8 Statistical analysis

Data were presented as means ± SEM. Statistical analysis was conducted using either paired or unpaired Student *t*‐tests, Mann–Whitney test, ordinary two‐way ANOVA or repeated measures two‐way ANOVA as detailed in figure legends. The significance level was set at *p* < 0.05.

## 3 RESULTS

### 3.1 HFD feeding disrupted the cardiorenal function in male mice

**FIGURE 1:**
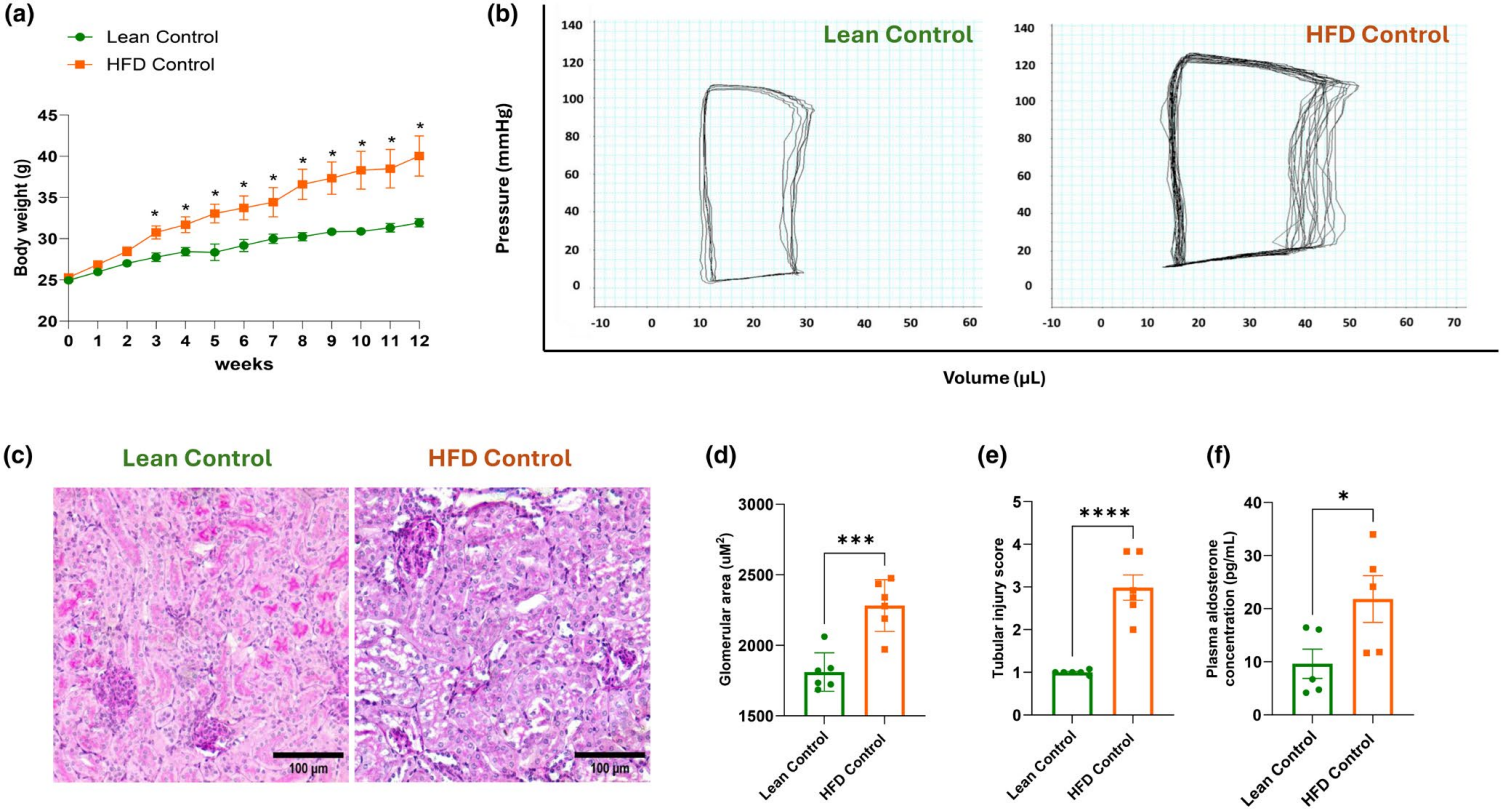
HFD feeding in mice impaired cardiac and renal function. Male mice were divided into two groups and fed either chow or a 60% HFD, with body weight monitored daily. (a) Body weight. (b) Representative pressure and volume loops. (c–e) Tubular injury scores and glomerular area were assessed using Periodic Acid‐Schiff (PAS) staining, quantified by ImageJ (bar scale: 100 μm). (f) Plasma aldosterone concentration. Statistical analysis was performed using repeated measures two‐way ANOVA (panel a, *N* = 8), unpaired Student's *t*‐test (panel d, *N* = 6; panel f, *N* = 5), and Mann–Whitney test (panel e, *N* = 6). *p < 0.05, ***p < 0.001, ****p < 0.0001. HFD, high‐fat diet.

HFD feeding in male mice for 16 weeks caused a significant increase in body weight when compared to lean control mice (Figure 1a). PV loop analysis was performed to assess cardiac function. The PV loops of HFD‐fed mice were taller, wider and shifted to the right when compared with those of lean controls (Figure 1b). Furthermore, HFD‐fed mice displayed a significant increase in systolic, diastolic and pulse pressures compared to lean controls (Table 1). End‐diastolic volume (Ved), end‐systolic and diastolic pressures (Pes and Ped, respectively) and stroke work (SW) were also increased in HFD‐fed obese mice. The hearts of HFD‐fed mice also underwent adverse remodelling, exhibiting cardiac fibrosis and cardiomyocyte hypertrophy as previously demonstrated,^22^ therefore, working under higher pressures and increased volumes. The HFD feeding also impaired diastolic function, with increased left ventricular relaxation time (Tau). All other PV loop parameters remained similar between HFD‐fed obese mice and lean control mice (Table 1).

**TABLE 1:** Hemodynamic parameters from the pressure–volume loop analyses of chow‐fed lean‐control mice and HFD‐fed obese mice (HFD‐control).

Parameters	Lean‐control	HFD‐control
Blood pressure parameters		
Systolic blood pressure (mmHg)	93.2 ± 2.39	106.5 ± 2.0**
Diastolic blood pressure (mmHg)	64.3 ± 1.6	72.4 ± 1.8**
Pulse pressure (mmHg)	29.0 ± 0.7	34.0 ± 0.7***
Baseline parameters		
Heart rate	554.4 ± 12.5	554.3 ± 8.7
End‐systolic volume (Ves) (μL)	16.1 ± 2.1	21.1 ± 1.5
End‐diastolic volume (Ved) (μL)	31.6 ± 1.7	47.6 ± 4.3**
End‐systolic pressure (Pes) (mmHg)	85.9 ± 1.9	106.1 ± 1.7****
End‐diastolic pressure (Ped) (mmHg)	6.3 ± 0.8	10.9 ± 1.2**
Stroke volume (SV) (μL)	22.9 ± 1.9	31.4 ± 4.0
Ejection fraction (EF) (%)	60.6 ± 2.4	61.9 ± 2.1
Cardiac output (CO) (μL/min)	13,443 ± 715.9	15,503 ± 1671
Stroke work (SW) (mmHg*μL)	2008 ± 138.8	2908 ± 363.8*
dP/dt max (mmHg/s)	8657 ± 267.7	9313 ± 312.2
dP/dt min (mmHg/s)	−8481 ± 127.7	−8879 ± 307.8
Tau (ms)	5.7 ± 0.2	6.5 ± 0.3*
IVC occlusion		
ESPVR (mmHg/μL)	4.3 ± 0.4	3.6 ± 0.5
EDPVR (mmHg/μL)	0.1 ± 0.01	0.1 ± 0.02
Ventricular‐arterial coupling (VAC)		
Arterial elastance (Ea) (mmHg/μL)	3.7 ± 0.3	3.9 ± 0.5
End‐systolic elastance (Ees) (mmHg/μL)	5.6 ± 0.9	5.0 ± 0.3
VAC index	0.6 ± 0.1	0.7 ± 0.1

*Note*: Values are mean ± SEM for *N* = 8 for lean‐control and HFD‐control. Unpaired Student *t*‐test was used for statistical analysis. *p < 0.05, **p < 0.01, ***p < 0.001, ****p < 0.0001 compared with lean‐control.

Abbreviations: EDPVR, end‐diastolic pressure volume relationship; ESPVR, end‐systolic pressure volume relationship.

We next examined how HFD feeding in mice affected kidney morphology and function. PAS staining revealed that HFD‐fed mice had enlarged glomerular area compared to those of lean control mice (Figure 1c,d). Furthermore, HFD feeding caused tubular injury, with the presence of tubular epithelial cell vacuolar deformation/hypertrophy, tubular deformation, tubular dilation, cell lysis and loss of the brush border in proximal tubules (Figure 1c,e). Interestingly, HFD‐induced cardiorenal remodelling was associated with an elevated plasma aldosterone concentration when compared to that in the lean controls (Figure 1f).

### 3.2 Eplerenone treatment for 30 days ameliorated obesity‐associated cardiac dysfunction

**FIGURE 2:**
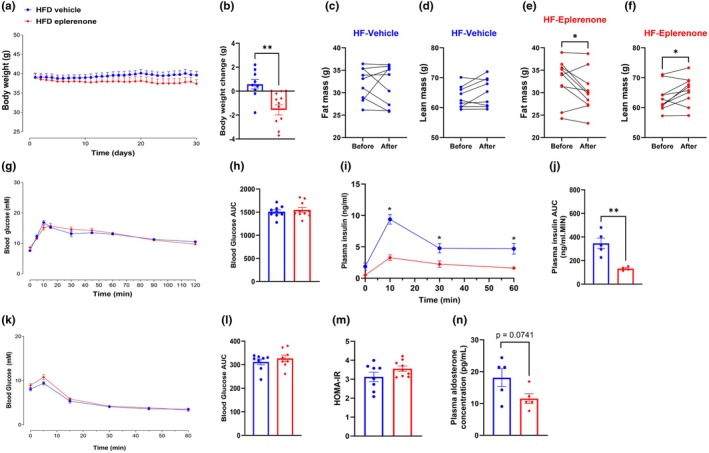
Effects of 30‐day administration of eplerenone on body weight, glucose tolerance and aldosterone. After 12 weeks of HFD feeding, male mice received either vehicle or eplerenone (200 mg/kg) for 30 days. (a) Body weight during treatment. (b) Body weight change between before and after treatments. (c–f) Fat and lean mass pre‐ and post‐treatment. (g) Blood glucose levels during the oral glucose tolerance test (OGTT) after treatment. (h) Area under the curve (AUC) for OGTT blood glucose. (i) Plasma insulin concentrations during OGTT. (j) AUC for OGTT insulin. (k) Blood glucose levels during the insulin tolerance test (ITT). (l) AUC for ITT blood glucose. (m) Homeostatic model assessment for insulin resistance (HOMA‐IR). (n) Plasma aldosterone concentration. Statistical analysis was performed using repeated measures two‐way ANOVA for panels a, g, i and k, paired Student's *t*‐test for panels c–f, and unpaired Student's *t*‐test for panels b, h, j, l, m, and n. *N* = 8–9 for all panels except for panels i, j and n, where *N* = 5. *p < 0.05, **p < 0.01. HFD, high‐fat diet.

To further examine the role of elevated plasma aldosterone or RAAS activation, obese mice were treated with eplerenone or vehicle control. Body weights were not significantly different between HFD‐fed mice treated with the vehicle (HFD vehicle) and those treated with eplerenone (HFD eplerenone; Figure 2a). While vehicle treatments did not significantly change body mass, fat mass or lean mass (Figure 2b–d), eplerenone caused a weight loss in obese mice (Figure 2b). This weight loss was mainly attributed to a loss in fat mass (Figure 2e). In contrast, eplerenone caused a mild but significant gain in lean mass (Figure 2f).

Furthermore, there was no difference in the oral glucose tolerance test (OGTT) between the groups after the treatments, as confirmed by the same area under the curves (AUC) (Figure 2g,h). Interestingly, plasma insulin levels during OGTT were significantly lower in HFD Eplerenone mice compared to HFD Vehicle mice, evidenced by lower AUC (Figure 2i,j). The insulin tolerance test (ITT) showed no differences between the groups (Figure 2k,l). The homeostatic model assessment of insulin resistance (HOMA‐IR) was not different between groups (Figure 2m). However, as expected, eplerenone treatment tended to reduce plasma aldosterone levels, albeit insignificant (Figure 2n; *p* = 0.0741).

**FIGURE 3:**
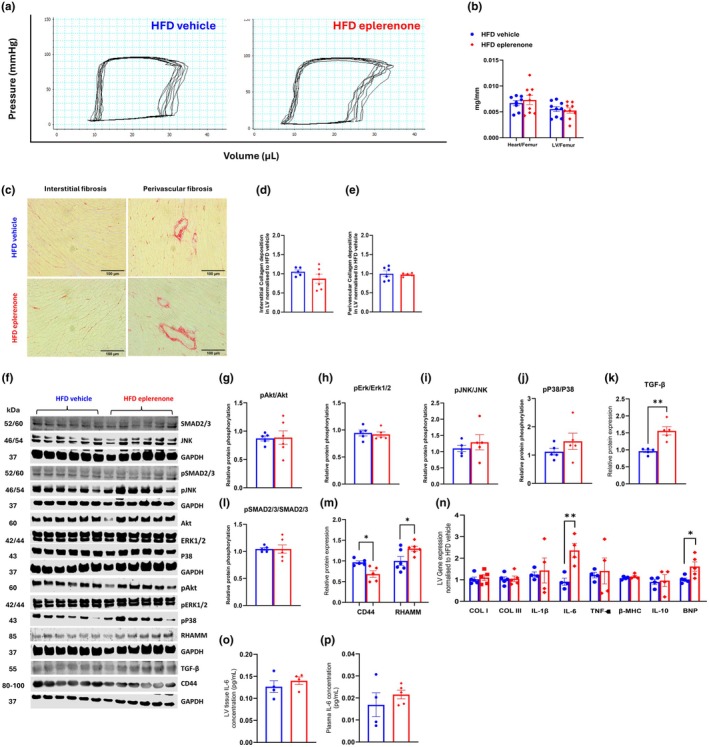
Eplerenone treatment ameliorated HFD‐induced cardiac dysfunction in obese mice. C57BL/6 mice were fed a 60% HFD. After 12 weeks of HFD feeding, mice received daily vehicle or eplerenone (200 mg/kg) for 30 days. Pressure–volume (PV) loop analysis was performed to assess cardiac function. (a) Representative PV loops. (b) Heart and left ventricle mass normalised to femur bone. (c–e) Interstitial and perivascular collagens detected by Sirius Red staining in left ventricles, quantified by ImageJ (bar scale: 100 μm). (f) Representative Western blots. (g–m) Quantitative Western blotting for pAkt/Akt, pErk/Erk1/2, pJNK/JNK, pP38/P38, TGF‐β, pSMAD2/3/SMAD2/3, CD44 and RHAMM. (n) qPCR quantification of mRNA expression, normalised to the HFD vehicle group. (o) Left ventricle IL‐6 concentration. (p) Plasma IL‐6 concentration. Statistical analysis was performed using unpaired Student's *t*‐test for all panels. (a, b) *N* = 8 for HFD vehicle, and *N* = 9 for HFD eplerenone; (c–p) *N* = 5–6. *p < 0.05, **p < 0.01, compared with HFD vehicle. HFD, high‐fat diet.

We further examined how eplerenone affected cardiac dysfunction in obese mice by PV loops (Figure 3a). Table 2 shows that eplerenone had no effect on blood pressures when compared to vehicle‐treated obese mice. However, there was a significant increase in Ped and ejection fraction (EF) in the HFD eplerenone mice compared with the HFD Vehicle mice. In contrast, eplerenone caused a significant reduction in stroke volume (SV), cardiac output (CO) and SW. Other cardiac parameters remained unchanged between the two groups. These findings suggest that eplerenone prevented HFD‐induced adverse myocardial remodelling in obese mice (e.g., decreased SV, SW, CO and increased EF).

**TABLE 2:** Hemodynamic parameters from the pressure–volume loop analyses of HFD‐fed mice receiving vehicle or eplerenone.

Parameters	HFD vehicle	HFD eplerenone
Blood pressure parameters		
Systolic blood pressure (mmHg)	106.5 ± 2.8	106.9 ± 2.4
Diastolic blood pressure (mmHg)	74.9 ± 2.2	76.1 ± 2.0
Pulse pressure (mmHg)	31.6 ± 0.9	31.2 ± 0.5
Baseline parameters		
Heart rate	577 ± 12.0	568.1 ± 6.4
End‐systolic volume (Ves) (μL)	12.6 ± 1.5	8.6 ± 1.9
End‐diastolic volume (Ved) (μL)	24.5 ± 1.5	20.4 ± 2.1
End‐systolic pressure (Pes) (mmHg)	88.8 ± 2.8	86.6 ± 3.8
End‐diastolic pressure (Ped) (mmHg)	7.6 ± 0.7	10.8 ± 0.8*
Stroke volume (SV) (μL)	18.0 ± 0.7	14.6 ± 0.6**
Ejection fraction (EF) (%)	62.3 ± 2.8	73.4 ± 4.0*
Cardiac output (CO) (μL/min)	10,989 ± 438.7	8611 ± 576.1**
Stroke work (SW) (mmHg*μL)	1604 ± 83.0	1283 ± 53.3**
dP/dt max (mmHg/s)	9885 ± 708.1	8764 ± 402.3
dP/dt min (mmHg/s)	−8622 ± 780.4	−7769 ± 355.4
Tau (ms)	5.4 ± 0.3	6.1 ± 0.3
IVC occlusion		
ESPVR (mmHg/μL)	8.7 ± 0.8	8.4 ± 0.9
EDPVR (mmHg/μL)	0.1 ± 0.01	0.1 ± 0.02
Ventricular‐arterial coupling (VAC)		
Arterial elastance (Ea) (mmHg/μL)	5.4 ± 0.3	5.2 ± 0.3
End‐systolic elastance (Ees) (mmHg/μL)	7.8 ± 0.8	10.1 ± 1.6
VAC index	0.7 ± 0.1	0.5 ± 0.1

*Note*: Values are mean ± SEM for *N* = 9 for HFD vehicle and *N* = 10 for HFD eplerenone. Unpaired Student's *t*‐test was used for statistical analysis. *p < 0.05, **p < 0.01 compared with HFD vehicle.

Abbreviations: EDPVR, end‐diastolic pressure volume relationship; ESPVR, end‐systolic pressure volume relationship.

### 3.3 Mechanistically, eplerenone‐induced improvement of cardiac function was associated with decreased CD44 and increased RHAMM and TGF‐β protein expression in the left ventricle

Despite improved cardiac performance in HFD Eplerenone mice, the wet weights of the heart or left ventricle were not affected by eplerenone (Figure 3b). Moreover, no significant changes in collagen deposition were observed between HFD Vehicle and HFD Eplerenone mice (Figure 3c–e). We next studied the role of eplerenone in regulating cellular signalling pathways that have been previously shown to be relevant to cardiac function. Eplerenone treatment did not affect Akt, Erk1/2, JNK or P38 phosphorylation in the left ventricle of mice (Figure 3f–j). Interestingly, eplerenone increased protein expression of TGF‐β, without affecting the canonical TGF‐β signalling mediated by SMAD2/3 phosphorylation (Figure 3f,k,l). Eplerenone treatment significantly reduced CD44 expression while increasing RHAMM expression in the left ventricle of HFD‐fed mice compared to HFD vehicle controls (Figure 3f,m). Moreover, eplerenone increased mRNA expression of IL‐6 and BNP, without affecting mRNAs of collagen I (Col I), collagen III (Col III), IL‐1β, TNF‐α, β‐MHC or IL‐10 (Figure 3n). However, increased IL‐6 mRNA did not correspond to increased IL‐6 concentration in the left ventricle of HFD eplerenone mice (Figure 3o). Moreover, plasma IL‐6 concentration was not different between HFD vehicle and HFD eplerenone mice (Figure 3p).

### 3.4 Eplerenone treatment for 30 days ameliorated obesity‐associated renal dysfunction

**FIGURE 4:**
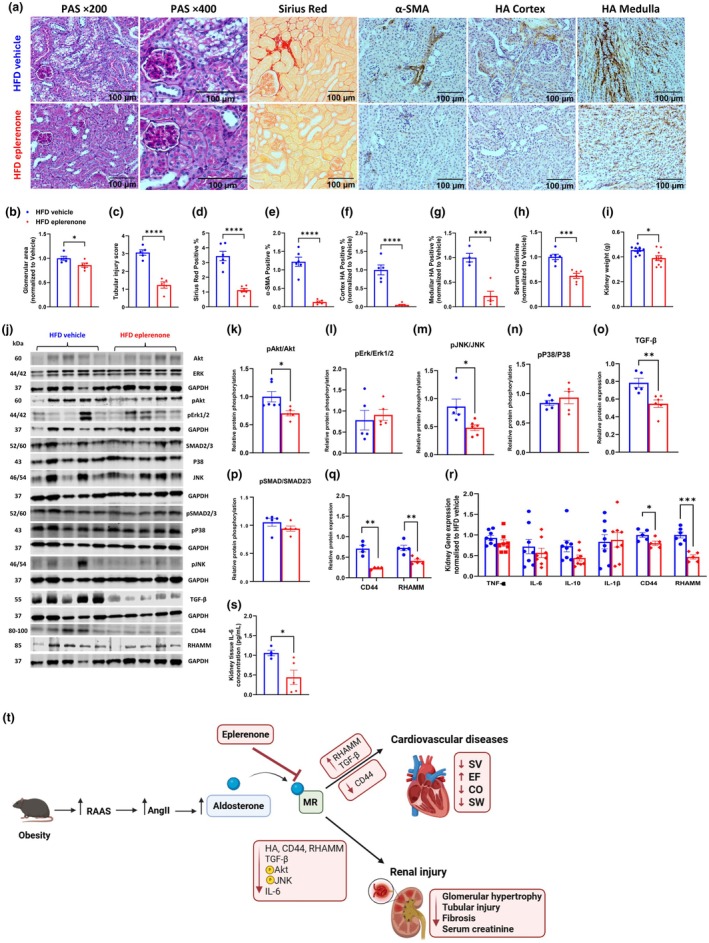
Eplerenone treatment ameliorated HFD‐induced renal dysfunction in obese mice. C57BL/6 mice were fed a 60% HFD. After 12 weeks of HFD feeding, mice received daily vehicle or eplerenone (200 mg/kg) for 30 days. At the end of the study, kidneys were collected postmortem for mechanistic studies. (a) Representative immunohistochemistry images. (b, c) Tubular injury scores and glomerular area assessed by Periodic Acid‐Schiff (PAS) staining. (d) Collagen deposition detected by Sirius Red staining. (e) α‐SMA (smooth muscle Actin) expression detected by immunohistochemistry. (f, g) Hyaluronan (HA) in the cortex and medulla detected by immunohistochemistry, quantified by ImageJ (bar scale: 100 μm). (h) Serum creatinine levels as a measure of kidney function. (i) Wet kidney weights. (j) Representative Western blots. (k–q) Quantitative Western blotting for pAkt/Akt, pErk/Erk1/2, pJNK/JNK, pP38/P38, TGF‐β, pSMAD2/3/SMAD2/3, CD44 and RHAMM. (r) qPCR quantification of mRNA expression, normalised to the HFD vehicle group. (s) Kidney tissue IL‐6 concentration. Statistical analysis was performed using unpaired Student's *t*‐test for all panels except that Mann–Whitney test was used for panel (c). *N* = 5–6 for all panels except for panels (i) and (r). Panel (i): *N* = 9–10; Panel (r): *N* = 5–6 for CD44 and RHAMM and *N* = 8 for all other genes. *p < 0.05, **p < 0.01, ***p < 0.001, ****p < 0.0001, compared with HFD vehicle. (t) Schematic illustration of eplerenone's protective effects against cardiac and renal dysfunction. Obesity activates the RAAS and increases Angiotensin II and aldosterone levels, leading to cardiac and renal dysfunction. By inhibiting aldosterone receptor signalling, eplerenone reverses cardiac dysfunction by increasing RHAMM and TGF‐β expression and decreasing CD44 expression. In contrast, in the kidney, eplerenone mitigates glomerular hypertrophy, tubular injury, inflammation and fibrosis, leading to decreased serum creatinine levels. Eplerenone does so via decreasing hyaluronan (HA) deposition, protein expression of CD44, RHAMM and TGF‐β, IL‐6, and phosphorylation of Akt and JNK. RAAS: Renin‐angiotensin‐aldosterone system. Ang II, angiotensin II; CO, cardiac output; EF, ejection fraction; MR, mineralocorticoid receptor; SV, stroke volume; SW, stroke work.

In the kidney, 30‐day eplerenone treatment significantly improved obesity‐associated morphological changes by decreasing glomerular area and tubular injury scores (Figure 4a–c). Eplerenone also ameliorated renal fibrosis by decreasing collagen deposition and protein expression of α‐SMA, a marker of myofibroblast activation (Figure 4a,d,e). Interestingly, eplerenone treatment decreased hyaluronan (HA) deposition both in the cortex and medulla area (Figure 4a,f,g). Collectively, these morphological improvements led to an improvement in renal function, evidenced by decreased serum creatinine levels (Figure 4h). The wet weight of kidneys was also significantly lower in mice treated with eplerenone compared with that of the vehicle‐treated mice (Figure 4i).

### 3.5 Mechanistically, eplerenone‐induced improvement of renal function was associated with decreased phosphorylation of Akt and JNK, as well as decreased expression of TGF‐β, IL‐6 and HA receptors, CD44 and RHAMM

Eplerenone treatment in obese mice significantly decreased phosphorylation of Akt and JNK, without affecting phosphorylation of Erk and P38 in the whole kidney lysates (Figure 4j–n). Interestingly, eplerenone significantly decreased protein expression of TGF‐β without affecting its canonical signalling mediated by phosphorylation of SMAD2/3 (Figure 4j,o,p). Consistent with reduced HA deposition, gene and protein expression of HA receptors, CD44 and RHAMM, were significantly decreased by eplerenone in the kidney (Figure 4j,q,r). However, mRNA levels of pro‐ and anti‐inflammatory cytokines, TNF‐α, IL‐6, IL‐1β or IL‐10, were not affected by eplerenone (Figure 4r). Despite unchanged IL‐6 mRNAs, IL‐6 concentration was significantly lower in the kidney of HFD eplerenone mice (Figure 4s). Taken together, these results suggest that eplerenone ameliorated kidney pathology and function by reducing collagen deposition and myofibroblast activation, inhibiting HA deposition and expression of HA receptors, CD44 and RHAMM, decreasing TGF‐β expression and IL‐6 concentration, and decreasing phosphorylation of Akt and JNK.

## 4 DISCUSSION

The current study revealed a beneficial role of eplerenone, an aldosterone antagonist and commonly used antihypertensive drug, in ameliorating obesity‐associated cardio‐renal dysfunction in mice, with novel cellular mechanisms (Figure 4t). HFD feeding in mice caused adverse myocardial remodeling and renal morphological changes, leading to impaired function and tissue damage. In the left ventricle, eplerenone mitigated obesity‐induced elevations in SV, SW and CO, and caused an increase in EF, accompanied by elevated TGF‐β and RHAMM expression, and decreased CD44 expression. In contrast, eplerenone had a more prominent role in reversing renal damage and dysfunction in obese mice. Its renal protective role was associated with reduced renal fibrosis, decreased activation of Akt and JNK, decreased expression of TGF‐β and IL‐6, and notably suppressed HA deposition and expression of HA receptors, CD44 and RHAMM. The potential link between the aldosterone receptor and the HA‐CD44/RHAMM pathway is novel and had not been previously reported.

HFD feeding in mice contributes to several detrimental effects and metabolic disruptions, including increased adiposity and body weight, insulin resistance, hypertension, cardiac, renal and liver dysfunctions, neuroinflammation, cognitive deficits and osteoarthritis.^23^, ^24^, ^25^, ^26^ Likewise, our study showed a noteworthy gain in body weight and a marked increase in arterial blood pressures in mice post HFD feeding. These results were consistent with previous reports,^27^, ^28^, ^29^ suggesting that obesity induced adverse myocardial remodelling with elevated volumes (Ved, SV albeit insignificant) and pressures (Ped and Pes) possibly due to increased afterload and preload. The fact that both SW and Tau were significantly elevated indicated that HFD feeding in mice caused impairment in cardiac relaxation possibly due to the heart muscle becoming stiffer from increased ECM deposition and fibrosis.^22^ Eplerenone treatment partially prevented HFD‐induced adverse myocardial remodelling by decreasing SV, SW and CO and increasing EF. These beneficial effects of eplerenone, however, did not seem to be associated with changes in cardiac stiffness as assessed by collagen deposition or inflammation mediated by pro‐inflammatory MAPK signalling pathways or expression of pro‐inflammatory cytokines TNF‐α, IL‐1β or IL‐6. Intriguingly, the beneficial cardiac effect of eplerenone was associated with decreased CD44 expression and increased RHAMM expression. CD44 and RHAMM are both HA receptors and have been shown to promote fibrosis,^30^ while aldosterone also possesses pro‐fibrotic effects.^31^ However, the direct regulation between aldosterone and CD44 and RHAMM expression remains to be studied.

HFD feeding leads to significant kidney damage in mice, affecting both tubular and glomerular structures. Mice on an HFD exhibited tubular injuries, including tubular dilation and atrophy, and glomerular enlargement, indicative of glomerular hypertrophy. These early kidney remodelling and damage are common in metabolic syndrome and obesity‐related nephropathy.^32^ Similar findings have been previously reported, showing that HFD feeding in mice increased lipid accumulation and promoted oxidative stress and inflammation in renal tissues, leading to glomerular hypertrophy and tubular injury.^33^ Additionally, HFD feeding elevates serum creatinine levels, indicating impaired renal function.^33^ Eplerenone treatment remarkably reversed these HFD‐induced renal damages. Eplerenone ameliorated glomerular hypertrophy, tubular injury, inflammation, fibrosis and activation of Akt and JNK pathways. Intriguingly, eplerenone suppressed HA deposition as well as gene and protein expression of HA receptors, CD44 and RHAMM. Despite the differential regulation on RHAMM, eplerenone decreased CD44 expression in both the kidney and left ventricle, suggesting the implication of CD44 and RHAMM in mineralocorticoid receptor antagonism.

Intriguingly, we observed that eplerenone increased TGF‐β in the left ventricle but decreased TGF‐β in the kidney of obese mice, without affecting its canonical signalling mediated by phosphorylation of SMAD2/3. Although TGF‐β as a therapeutic target is still debatable, delivery of mRNA TGF‐β has shown therapeutic effects on type 1 diabetes mellitus, experimental allergic encephalomyelitis, arthritis, systemic lupus erythematosus (SLE) and inflammatory bowel disease.^34^, ^35^, ^36^ It has been reported that TGF‐β enhances the healing process after a myocardial infarction. TGF‐β also protects cardiomyocytes from ischemic injury.^34^, ^37^, ^38^ However, its role beyond the canonical SMAD2/3 signalling in the heart and kidney, as well as under eplerenone treatment, remains to be elucidated. It is also worth mentioning that the renoprotective effect of eplerenone was accompanied by a significant reduction in renal IL‐6 concentration, yet IL‐6 levels in the left ventricle or in the circulation were not affected. This tissue‐specific effect of eplerenone on IL‐6 is interesting but warrants further investigations.

It is possible that the cardio‐renal protective role of eplerenone resulted from a reduced body weight and an improved body composition (decreased fat mass and increased lean mass). It is known that eplerenone can also affect body fluid balance. However, our body composition measurement by EchoMRI did not capture the measurements on total body water; therefore, the potential impact of eplerenone on fluid balance is unknown. Wada et al. have previously shown that eplerenone prevented excessive body weight gain and fat accumulation, ameliorated glucose and insulin intolerance, and enhanced energy expenditure in HFD‐fed mice.^39^, ^40^ While our results were consistent with findings by Wada et al. on decreased body weight gain and fat accumulation, we did not observe any differences in glucose or insulin tolerance with eplerenone. Instead, we observed decreased insulin concentrations during the glucose tolerance test in HFD‐fed eplerenone‐treated mice. These results suggest that eplerenone might impair glucose‐stimulated insulin secretion and/or improve insulin sensitivity, given the lower plasma insulin levels but maintained glucose tolerance. However, it is acknowledged that insulin tolerance is a test measuring whole‐body glucose clearance in response to a bolus of exogenous insulin but not a precise method for measuring insulin sensitivity. Insulin concentration during a glucose tolerance test is a readout of the difference between glucose‐stimulated insulin secretion and insulin clearance. Therefore, the exact role of eplerenone on insulin sensitivity and insulin secretion remains to be determined.

Although eplerenone is a hypertensive medication,^41^ we found that it did not affect arterial blood pressure in HFD‐fed obese mice in the current study. This could be attributed to the mild effect of HFD on arterial blood pressures relative to other hypertensive models such as salt‐sensitive hypertensive rats, spontaneously hypertensive rats (SHR), and WKY rats.^42^, ^43^ In fact, HFD feeding in mice only mildly elevated arterial blood pressures, with the absolute values remaining within the normotensive range and below the defined threshold for hypertension.^44^, ^45^ Moreover, it is worth highlighting the dramatic differences in the PV loop parameters in HFD‐control (Table 1) and HFD vehicle (Table 2). While studies in Tables 1 and 2 were performed at different times by different researchers, several technical and physiological factors could have contributed to these variations, including varying sizes of the PV catheters subject to catheter availability and operator preference, varying duration of anaesthesia due to operator difference, and the presence of vehicle treatment. However, the oral saline vehicle was administered in small volumes and was unlikely to have influenced preload or haemodynamic parameters. Anaesthesia has been shown to profoundly influence cardiovascular measurements in mice, typically reducing blood pressure, heart rate and cardiac output compared with conscious states. For instance, isoflurane (1–2%) can lower mean arterial pressure by 10–20 mmHg through sympathetic inhibition.^46^, ^47^, ^48^ Nevertheless, all animals underwent identical PV loop procedures; thus, any anaesthesia‐related effects were equivalent and should not compromise the conclusion of the study.

While eplerenone produced clear cardiorenal protective effects in our model of obesity, we acknowledge the absence of parallel improvements in systemic blood pressure and whole‐body insulin tolerance. This divergence is supported by preclinical studies suggesting that mineralocorticoid receptor antagonism can confer organ‐specific benefits independent of systemic haemodynamic or metabolic changes. For example, in a murine model of HFD‐induced renal injury, eplerenone reduced albuminuria and improved kidney morphology without significantly affecting systolic blood pressure or serum creatinine levels.^49^ Additionally, studies in diabetic rodents have demonstrated that eplerenone attenuates renal inflammation and structural damage, even in the absence of changes in blood pressure or glycaemia.^50^ Moreover, mineralocorticoid receptor blockade in adipose tissue reduces obesity‐related insulin resistance by decreasing oxidative stress and inflammatory macrophage infiltration, yet these local improvements may be masked when evaluating systemic insulin sensitivity using ITT or GTT.^51^ Thus, the absence of systemic effects in our model likely reflects the localised actions of eplerenone, offering targeted renal protection through anti‐inflammatory and anti‐fibrotic pathways, while leaving broader metabolic and haemodynamic mechanisms of obesity, such as sympathetic activation, adipokine imbalance and insulin resistance, unaffected in the short term.

Our study exclusively used male mice, which are more prone to obesity‐induced cardiometabolic dysfunction, including robust visceral adiposity, insulin resistance and RAAS activation, compared with females. Pre‐menopausal females are partially protected by oestrogen from metabolic and inflammatory insults. Given the sexual dimorphism in RAAS signalling, the absence of female cohorts is a limitation that may affect the generalisability of our findings.

In conclusion, the findings of our study show beneficial effects of eplerenone in ameliorating obesity‐associated cardiac and renal dysfunction with novel cellular mechanisms. These results offer valuable insights into the potential therapeutic applications of eplerenone in obesity‐related cardio‐renal complications. We demonstrate for the first time that eplerenone can regulate cardiac and renal function by modulating IL‐6, TGF‐β and the HA and CD44/RHAMM pathway. These novel mechanistic insights advance our understanding of the mode of action of eplerenone, therefore providing evidence for future development of similar drugs or repurposing eplerenone for the treatment of obesity‐related complications.

## AUTHOR CONTRIBUTIONS

AB and LK contributed to the concept and experimental design, researched data, contributed to discussion and data interpretation and wrote the manuscript. BQ, RK, AL, TW, VM, JM and CM researched data, contributed to discussion and data interpretation and reviewed and edited the manuscript. All authors approved the final version of this manuscript. The authors received no editorial assistance. LK is the guarantor of this work and, as such, had full access to all the data in the study and takes responsibility for the integrity of the data and the accuracy of the data analysis.

## FUNDING INFORMATION

This work was supported by the British Heart Foundation (PG/18/56/33935 to LK) and Diabetes UK (15/0005256 and 21/0006329 to LK). AB was supported by the Kingdom of Saudi Arabia, Ministry of Education, Umm Al‐Qura University, BQ was supported by the China Scholarship Council and AL was supported by a Diabetes UK PhD studentship (22/0006477).

## CONFLICT OF INTEREST STATEMENT

There were no potential conflicts of interest relevant to this article.

## Data Availability

The data that support the findings of this study are available from the corresponding author upon reasonable request.
